# Alpha diversity with increasing altitude and Rapoport’s rule adherence: Elevational trends in Noctuoidea (Lepidoptera) of Mizoram, India

**DOI:** 10.3897/BDJ.13.e152977

**Published:** 2025-04-25

**Authors:** Malsawmtluanga Hnialum, Chitra Narayanasami, Santosh Ganapati Patil, Soundararajan Raga Palanisamy, Kumaraperumal Ramalingam, Balasubramani Venkatasamy, Amrit Sekhar Mallick, Dilipsundar Natarajan, Arulkumar Gopal, Lalmuanzuala B

**Affiliations:** 1 Tamil Nadu Agricultural University, Coimbatore, India Tamil Nadu Agricultural University Coimbatore India

**Keywords:** alpha diversity, biogeography, elevational gradient, Noctuoidea, Rapoport’s rule, Indo-Myanmar

## Abstract

An assessment of the alpha diversity across the altitudinal range sizes for the moths of Noctuoidea (Lepidoptera) collected from 25 locations in Mizoram, a biodiversity hotspot in India, was undertaken. A total of 164 moth specimens were examined. based on morphological and genitalia characteristics for their identity. Identified specimens belonged to 42 species and 34 genera of three families Erebidae, Noctuidae and Nolidae. The results showed that the alpha diversity of Noctuoidea moths was the highest at the lower middle altitude range (400-800 m), while the least was at the lower elevation range (0-400 m). In contrast, the species’ ranges increased with rising altitude. Steven’s (R = 0.903, p < 0.001), Pagel’s (R² = 0.873, p < 0.06), Rohde’s (R² = 0.961, p < 0.001) and cross-species (R² = 0.013, p < 0.6) methods were used to test Rapoport’s rule. They yielded three positive results with the cross-species method being negative due to outliers in the species distribution. Rapoport’s rule applicability for moths in the Indo-Myanmar biodiversity hotspot was evaluated for the first time. These findings are vital in explaining the elevational diversity patterns of noctuoid moths in northeast India and Indo-Myanmar, leading to a better understanding of the region's biogeography.

## Introduction

Noctuoidea (Lepidoptera) comprises approximately 43,000 known species with over 3,800 genera with many more to be discovered (van Nieukerken et al. 2011). Noctuoidea includes Erebidae, Eutellidae, Noctuidae, Nolidae, Notodontidae and Oenosandridae. Of these, some Noctuoidea larvae (armyworms, cutworms, semiloopers and hairy caterpillars) are serious agricultural pests, viz., larvae completely devastate the crops as defoliators, inflorescence feeders and fruit borers. Furthermore, adults of some noctuoids are also pests of crops, such as fruitsucking moths that feed on tomatoes, citrus fruits, pomegranates etc. (Metcalf and Flint 1993). However, these Noctuoids are also important pollinators (Banziger 1982, Kitching 1984) and play an important role in the food web as a source of sustenance to various faunal species (New 2023). Approximately 6,000 noctuoid species are deemed commercially relevant (Zhang 1994) and these moths are found in all major zoogeographic areas worldwide.

Species diversity varies with elevation due to numerous factors such as climatic variation, habitat heterogeneity, species dispersal abilities, farming and urbanisation of the ecosystems (Beck et al. 2016a, Chen et al. 2022). Elevational diversity studies also help in identifying areas of high primary productivity, indicating more significant overall biodiversity in plants in the elevation band corresponding to the area of higher species abundance and richness (Evans et al. 2005). Therefore, the study of diversity along an elevation gradient will hone the knowledge of researchers, relevant academic bodies and policy-makers to understand species range and its particular habitat which will ultimately aid in forming conservation plans (Quintero and Jetz 2018).

The impact of geographical features like altitude, latitude and depth on species distribution is important as it reflects the distribution of species in different biogeographical ranges. According to Rapoport’s rule, species' geographic ranges positively correlate with their relative position in a gradient such as latitude, altitude or depth (Rapoport 1982, Stevens 1989). This pattern is called the Rapoport's effect or rule and is often attributed to climatic variability, competition and environmental filtering (Pintor et al. 2015). This rule has been widely tested in vertebrates, plants and insects in both terrestrial and aquatic ecosystems (Stevens 1989, Stevens 2003, Morin and Chuine 2006, Liu et al. 2020). There has been a handful of times when there was a negative Rapoport’s rule, such as in the case of Bhattarai and Vetaas (2006) where the most expansive range was found in the middle elevation bands. There is only one study of Rapoport’s rule in India concerning lepidopterans which assessed the range size of butterflies in the eastern Himalayas (Dewan and Acharya 2024).

Mizoram (21°58'–24°35' N and 92°15'–93°29' E) is the easternmost State of India, bordering Bangladesh to its southwest and Myanmar to its east. The elevation ranges from 0 to 2,157 m above sea level, but averages at about 500 m to 800 m. It is considered a part of the ‘Manipur-Kachin Rain Forests Ecoregion (Sati 2023). Mizoram receives very high annual rainfall (> 2500 mm) (Monsang et al. 2021), which varies with the specific location and elevation. Owing to its location in the Indo-Myanmar biodiversity ecoregion, the north-eastern Indian State of Mizoram is an example of the region's rich biodiversity (Solanki et al. 2024). According to the Zoological Survey of India (Ramakrishna 2007), Mizoram has > 1440 species of fauna, belonging to 891 genera of 295 families. Insects constitute 520 species, birds with approximately 380 species, 96 mammalian species, 505 avian species, 117 reptile species, 60 amphibian species and 142 fish species (Sati 2023). A few lepidopteran species, including endangered ones in central Nepal, are also found in Mizoram and the surrounding areas, demonstrating the region's influence from the Eastern Himalayan biodiversity hotspot (Khanal et al. 2015). Despite the region being a known hotspot, there is a dearth of scientific research on moths in this region.

The present study investigates how the alpha diversity of moths from the superfamily Noctuoidea varies along an elevational gradient in Mizoram and whether a positive Rapoport effect is observed in this moth family. By analysing species richness and elevational range sizes, we seek to contribute to the broader understanding of insect biogeography and biodiversity in mountainous ecosystems of India.

## Material and methods

### Collection and Identification

**Figure 1.**
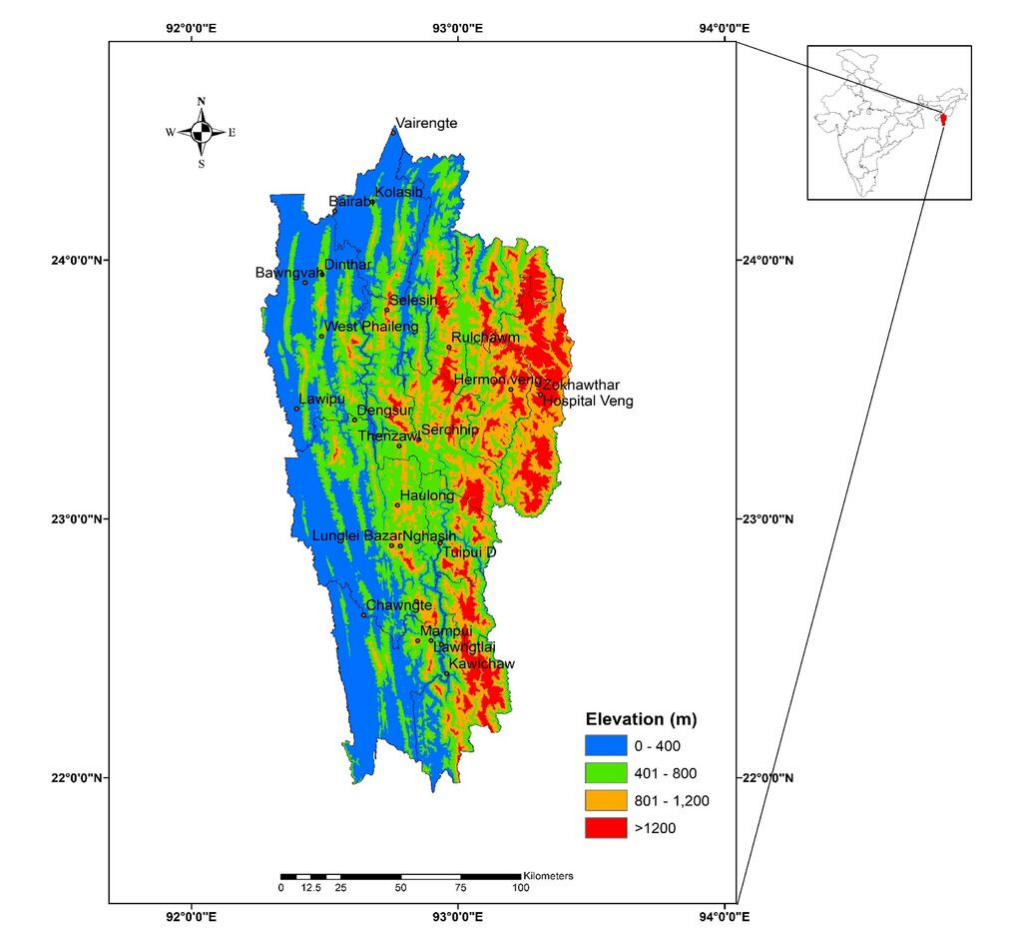
Map of Mizoram with surveyed locations marked.

Noctuoidea moths were collected in 25 locations in different altitudes across Mizoram (Fig. 1,Table 1). Samples were collected using a light trap (160 watt mercury vapour lamp) and a white sheet of cloth 3.048 × 4.572 metre (m). The collected specimens were identified, based on their morphological characters, then the noctuoid moths were separated and identity was further confirmed up to the genus with genitalia examination (Robinson 1976). Photographs of specimens were taken with Canon EOS 5D Mark IV attached to a macro lens (Canon Macro 100mm lens) and genitalia were observed using a Leica M205A stereomicroscope with the Leica LAS X Software (Version 5.2.2).

### Alpha Diversity Analysis

The species richness of noctuoid moths along the different elevation ranges was made using asymptotic diversity, based on Hill number q (Hill 1973, Chao et al. 2014). Species richness (q = 0), Shannon diversity (q = 1) and Simpson’s diversity (q = 2) were calculated along with extrapolation, based on the number of individual species to be sampled (Dataset available in Suppl. material 1). The presence of species was estimated with species richness (q = 0), the diversity estimation was done using Shannon diversity index (q = 1) and the abundance of each species was estimated to account for common species in the samples. The dominant species and its number in the community were estimated using Simpson’s diversity (q = 2). All the analyses were performed using a resampling bootstrap of 1000 and a confidence level of 95% (Chao et al. 2020).

### Rapoport’s rule analysis

Rapoport’s rule was tested in this study using Steven’s, Pagel’s, Rohde’s and cross-species methods due to inconsistencies found in single method studies (Böhm et al. 2017). The elevation of the study area is divided into four levels, based on their vegetation type, each 400 m in range. The mean altitudinal range of each species was calculated, based on the mean elevation of each sampled specimen for Steven’s method (Stevens 1989). In Rohde’s method, each species was designated as an independent unit from which the mid-point between the lowest and highest elevation record was calculated (Rohde et al. 1993). Pagel's method quantified the average species range of all the species whose upper distribution limit fell within a given latitudinal band (Pagel et al. 1991). In the cross-species method, a scatter-plot was made with an altitudinal range size and the mid-point of each species as coordinates (Letcher and Harvey 1994). The slopes of the four models were obtained by fitting a linearity test to prove adherence to Rapoport’s Rule (Liu et al. 2020).

### Statistical analysis

All the statistical analyses were performed in Rstudio (2023.12.1 Build 402) with the r package iNext (Hsieh et al. 2016) and ggplot2 (Wickham 2011).

## Results

### Species Delimitation

A total of 164 individuals of Noctuoid moths were identified. They were classified under three families viz. Erebidae, Noctuidae and Nolidae. Erebidae had the highest species richness (33) and abundance (146), with six subfamilies, 26 genera and 33 species making up 89% of all the collected samples. Noctuidae had four subfamilies, six genera and six species with 16 specimens which Nolidae followed with one subfamily, one species and two specimens (Suppl. materials 4, 5, 6, 7, 8, 9). Identification of species is the first step of accurate and precise diversity analysis (Armstrong and Ball 2005). An assemblage of 42 species were identified, based on their morphological appearance and their genital characteristics, which are strongly conserved within species, but typically diverge significantly after speciation (Gangotia et al. 2024). Many more moths were collected, but, due to damage to their morphology or abdomen, identification was challenging. The occurrence dataset associated with this study is available on GBIF (Global Biodiversity Information Facility) at Hnialum (2025).

### Alpha diversity along the elevation

**Figure 2.**
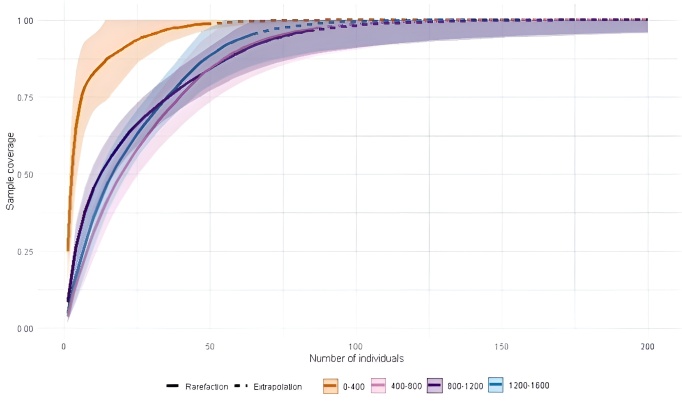
Sample completeness curve of the superfamily Noctuoidea in different elevation ranges of Mizoram.

The sample completeness curve (Fig. 2) results revealed that the sample coverage of each elevation range reached over 90% and the line flattened out, indicating sufficient sampling and suitability for further biodiversity analysis (Chen et al. 2022). The lowest elevation range (0–400 m) reached nearly 97.96% coverage, suggesting either a less diverse community or one that has been thoroughly sampled. The mid-elevation ranges (400–800 m and 800–1200 m) demonstrated high sample completeness, approaching 95.36% and 95.22%, respectively, ensuring a well represented dataset for these zones. The highest elevation range (1200–1600 m) exhibited a slightly lower sample coverage of approximately 91.69%, indicating a marginally lower completeness, but still sufficient for reliable biodiversity assessment (Suppl. material 10).

**Figure 3.**
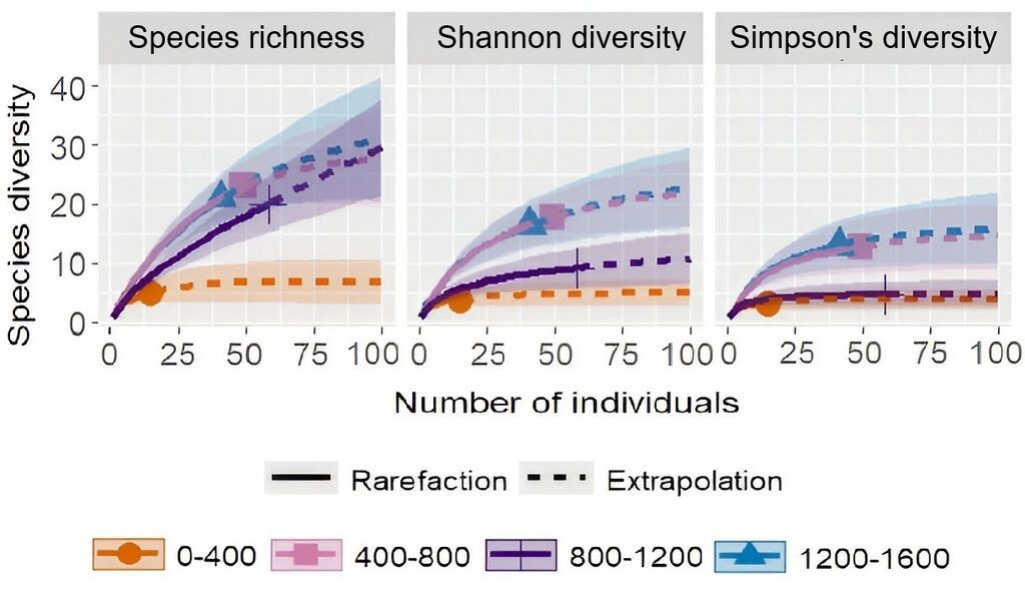
Sample-size-based rarefaction (solid line) and extrapolation (dotted line) sampling curves with 95% confidence intervals (shaded areas) of four different elevations for Species richness, Shannon diversity and Simpson's diversity, right panel.

The Hill number of the various elevations showed that the highest species richness (q = 0) was observed in the 400-800 m range (23) followed by 800-1200 m (21) and 1200-1600 m (21) ranges with the same richness value and the lowest richness was observed in the range of 0-400 m (5). The Shannon diversity values (q = 1) were the highest in the range of 400-800 m (19.7), followed by 1200-1600 m (17.6) then 800-1200 m and lastly 0-400 m (3.9). Simpson's diversity (q = 2) also showed the same trend with species richness with the highest Simpson's diversity found in 400-800 m (16.79) followed by 800-1200 m (14.69) then 1200-1600 m (9.99). The lowest Simpson’s diversity was observed in the 0-400 m (3.35) range (Fig. 3, Suppl. material 3). The most common species of Erebidae were *Miltochristaobliquilinea* (Arctiinae) and *Cyanaobliquilineata* (Arctiinae), while in Noctuidae, it was *Chrysodeixiseriosoma* (Plusiinae). The most dominant species were *C.obliquilineata* and *M.obliquilinea* both accounting for 10% of the total individuals, respectively with *C.obliquilineata* being more dominant in higher elevations (> 800 m) and *M.obliquilinea* being more numerous in lower elevations (< 800 m).

### Distribution Ranges and Rapoport’s Rule Test

The species with the least overall distribution range was found to be *Numenessiletti* (Lymantriinae) (62 m), while *Creatonotostransiens* (Arctiinae) (1556 m) and *M.obliquilinea* (Arctiinae) (1556 m) had the greatest distribution range. The mean elevational distributional range of all the specimens was 552.96 m when weighed, based on their number of occurrences.

**Figure 4.**
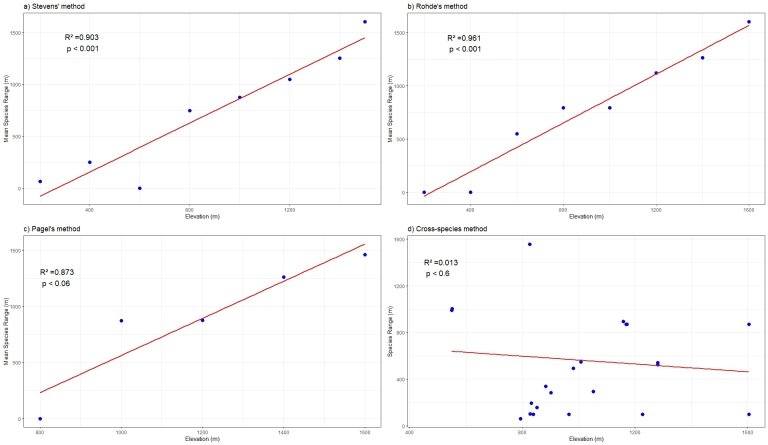
Rapoport’s rule test: **a** Stevens’ method; **b** Rohde’s method; **c** Pagel’s method; **d** cross-species method.

Three of the four tests for Rapoport’s rule were positive (Fig. 4, Suppl. material 2). Stevens' test showed a gradual increase in the altitudinal range with an increase in elevation which showed a positive linear relationship (R = 0.903, p < 0.001). The results of Rohde’s method showed that the mean species range followed a strong linear upward trend with increasing elevation. Across all elevations (0 m to 1600 m), the species range increased consistently, reaching its highest value at the highest elevation. The statistical results (R² = 0.961, p < 0.001) confirmed a highly significant correlation. Pagel’s method showed that the mean species range exhibited an increasing trend with altitude. The lowest mean species range occurred at lower elevations, while higher altitudes displayed a linear increase in species range. The linear regression line (R² = 0.873, p < 0.06) indicated a strong correlation, though the statistical significance is marginal. The cross-species method showed no clear relationship between species range and elevation. Though there was the formation of the distinct pyramid shape of the scatter-plot, various outlier data points led to the inability to establish a correlation. The regression line (R² = 0.013, p < 0.6) exhibited a weak downward trend with increasing altitude, but the high variability amongst data points shows no meaningful pattern.

## Discussion

### Alpha diversity along the elevation

The research findings revealed a peak of alpha diversity on the lower mid-elevation (400-800 m) (Fig. 3), which is consistent with findings observed in Geometridae (Lepidoptera) (Beck et al. 2016b). After this, there was a gradual decrease in the diversity on the elevation increase in line with that observed for family Geometridae (Chen et al. 2007) and Crambidae (Fiedler et al. 2008, Chen et al. 2022). The low number of specimens recorded in the lower elevation (0-400 m) may be attributed to light pollution and deforestation, as significant anthropogenic developments like urbanisation and laying out of new roads are more concentrated in the lower elevations (Hu et al. 2019, Wagner 2020). A study of trees in Sikkim, located in the northeast Indian region (Acharya et al. 2011), showed that tree species richness followed a hump-shaped relationship with elevation showing a peak at around 1500 m, which is likely the cause of the low observed moth diversity at the lowest and highest elevation. Various authors also note this mid-peak in tree diversity (Behera and Kushwaha 2006, Yanjun et al. 2020). This likely results in moths with a specialist diet being unable to inhabit areas where their host are rare or unavailable, which is more likely in the areas with lower tree diversities.

### Rapoport’s rule

In our study, Stevens’ method, Rohde’s method and Pagel’s method all showed positive linear results, indicating that the increase in elevation resulted in the gradual reduction of diversity and an increase in the habitat range of each species (Rapoport 1982). The cross-species method, however, did not show a positive result, which may be attributed to the outliers where some species have narrow ranges at high altitudes, while others may have broad ranges at lower altitudes, which resulted in a negative trend with no significant correlation on the regression line. The findings align with the Ambient Energy Hypothesis, which proposes that species at higher altitudes tend to have broader climate tolerances than those at lower altitudes (Stevens 1989, Wang et al. 2009). This may explain the deviation from other similar studies (Wang et al. 2009, Chen et al. 2022), where the absence of a substantial mid-domain effect and the straight positive correlation in Rohde’s method instead of a parabolic regression line that could be attributed to the study being limited to a lower elevation of 1600 m (Wang et al. 2009).

The small sample size along the altitudinal gradient may have influenced the predictions of Rapoport’s rule across different methods, the results of Stevens’ method, Rohde’s method and Pagel’s method indicating that the altitudinal ranges of species in Mizoram exhibit a positive correlation with elevation, with higher coefficients providing a theoretical foundation for understanding the altitudinal distribution patterns of Lepidoptera in this region and a scientific basis for large-scale biodiversity conservation.

## Conclusion

Given the limited studies on Lepidoptera diversity in Mizoram and the broader Indian area of the Indo-Myanmar biodiversity hotspot, this research provides a critical baseline for future work. The analysis of Rapoport’s Rule revealed a general positive correlation between elevation and species range size. These findings reinforce the importance of climatic tolerance and environmental filtering in determining moth distribution along an altitudinal gradient and support the general predictions of Rapoport’s rule.

## Supplementary Material

3FB1B888-1590-5187-ABFF-690D65254ABF 10.3897/BDJ.13.e152977.suppl1 Supplementary material 1 Dataset used in iNext Data type Excel sheet Brief description It is the dataset used in iNext for the calculation of the different biodiversity data and Rapoport's rule analysis. File: oo_1246409.xlsx https://binary.pensoft.net/file/1246409 Malsawmtluanga Hnialum, Chitra N, Balasubramani V, Soundararajan R.P, Kumaraperumal R, Patil Santosh Ganapati, Amrit Sekhar Mallick, Dilipsundar Natarajan

D5B8C0BA-BEF7-5C03-BFA9-CCB1270D94BA 10.3897/BDJ.13.e152977.suppl2 Supplementary material 2 Data acquired from Rapoports rule analysis Data type Excel sheet Brief description Results of Rapoports rule analysis. File: oo_1246411.xlsx https://binary.pensoft.net/file/1246411 Malsawmtluanga Hnialum, Chitra N, Balasubramani V, Soundararajan R.P, Kumaraperumal R, Patil Santosh Ganapati, Amrit Sekhar Mallick, Dilipsundar Natarajan

47B10368-E370-5CEC-94C2-B7D6C8A21DA7 10.3897/BDJ.13.e152977.suppl3 Supplementary material 3 Species richness, Shannon diversity and Simpson's diversity Data type Excel sheet File: oo_1246412.xlsx https://binary.pensoft.net/file/1246412 Malsawmtluanga Hnialum, Chitra N, Balasubramani V, Soundararajan R.P, Kumaraperumal R, Patil Santosh Ganapati, Amrit Sekhar Mallick, Dilipsundar Natarajan

2024EFBE-C90B-5071-B635-343918BD1557 10.3897/BDJ.13.e152977.suppl4 Supplementary material 4 Specimens collected record Data type Excel sheet File: oo_1246413.xls https://binary.pensoft.net/file/1246413 Malsawmtluanga Hnialum, Chitra N, Balasubramani V, Soundararajan R.P, Kumaraperumal R, Patil Santosh Ganapati, Amrit Sekhar Mallick, Dilipsundar Natarajan

BC3DFECF-45F9-586C-B894-BA140DB5393B 10.3897/BDJ.13.e152977.suppl5 Supplementary material 5 Plates of specimen collected 1 Data type JPG Brief description a) *Barsinedefecta* b) *Miltochristaphaeodonta* d) *Barsineroseata* e) *Creatonotostransiens* e) *Cyanaobliquilineata* f) *Cymeeuprepoides* g) *Dichromiaquadralis* h) *Hypenarhombalis*. File: oo_1246419.JPG https://binary.pensoft.net/file/1246419 Malsawmtluanga Hnialum, Chitra N, Balasubramani V, Soundararajan R.P, Kumaraperumal R, Patil Santosh Ganapati, Amrit Sekhar Mallick, Dilipsundar Natarajan

D6C6CFAE-9AB1-5887-87F3-C46F852B87DB 10.3897/BDJ.13.e152977.suppl6 Supplementary material 6 Plates of specimen collected 2 Data type JPG Brief description a)*Hypocalasubsatura* b)*Imausmunda* c)*Juxtarctiamultiguttata* d)*Miltochristaconjunctana* e)*Syntomoidesimaon* f)*Miltochristaobliquilinea* g)*Miltochristajarawa* h)*Neocherainops*. File: oo_1246417.JPG https://binary.pensoft.net/file/1246417 Malsawmtluanga Hnialum, Chitra N, Balasubramani V, Soundararajan R.P, Kumaraperumal R, Patil Santosh Ganapati, Amrit Sekhar Mallick, Dilipsundar Natarajan

47A99446-43AB-5ABF-B4D9-0282DD7CAB82 10.3897/BDJ.13.e152977.suppl7 Supplementary material 7 Plates of specimen collected 3 Data type JPEG Brief description a)*Ammatodivisa* b)*Ammathonavneetsinghi* c)*Ammathopseudodorians* d)*Arnabipunctapex* e)*Artaxadigramma* f)*Artaxaguttata* g)*Asotacaricae*. File: oo_1247290.JPG https://binary.pensoft.net/file/1247290 Malsawmtluanga Hnialum, Chitra N, Balasubramani V, Soundararajan R.P, Kumaraperumal R, Patil Santosh Ganapati, Amrit Sekhar Mallick, Dilipsundar Natarajan

D53CE019-36E7-5A4F-9A1C-DC6FEF050060 10.3897/BDJ.13.e152977.suppl8 Supplementary material 8 Plates of specimen collected 4 Data type JPEG Brief description a)*Nephelomiltaeffracta* b)*Miltochristavelona* c)*Orvascasubnotata* d)*Vamunamaculata* e)*Spilosomaobliqua* f)*Numenessiletti* g)*Thyascoronata* h)*Tinoliusquadrimaculatus* i)*Trigonodeshyppasia*. File: oo_1247295.JPG https://binary.pensoft.net/file/1247295 Malsawmtluanga Hnialum, Chitra N, Balasubramani V, Soundararajan R.P, Kumaraperumal R, Patil Santosh Ganapati, Amrit Sekhar Mallick, Dilipsundar Natarajan

9F3AEF61-E3BD-5E29-8C92-8992BBD6B269 10.3897/BDJ.13.e152977.suppl9 Supplementary material 9 Plates of specimen collected 5 Data type JPEG Brief description a)*Axyliaputris* b)*Callopistriaindica* c)*Chrysodeixiseriosoma* d)*Ctenoplusiatarassota* e)*Helicoverpaarmigera* f)*Spodopteralitura* g)*Thysanoplusiaorichalcea* h)*Thysanoptyxsordida*. File: oo_1247297.JPG https://binary.pensoft.net/file/1247297 Malsawmtluanga Hnialum, Chitra N, Balasubramani V, Soundararajan R.P, Kumaraperumal R, Patil Santosh Ganapati, Amrit Sekhar Mallick, Dilipsundar Natarajan

A2779300-97E8-5269-8EED-507C7CA447E2 10.3897/BDJ.13.e152977.suppl10 Supplementary material 10 Sample coverage Data type CSV data sheet Brief description The numerical results of sample coverage from iNEXT package. File: oo_1297277.csv https://binary.pensoft.net/file/1297277 Malsawmtluanga

## Figures and Tables

**Table 1.** Details of surveyed locations in Mizoram and types of vegetation.

**Sl. No**	**Altitude** **(m)**	**Location**	**Latitude and longitude**	**Type of Vegetation**	**Common Flora**
1	0-400	Bawngvah	23°91'26"N; 92°42'56"E	Tropical Evergreen and Semi-evergreen Forests	*Dipterocarpusturbinatus, Micheliachampaca, Artocarpus* spp., *Terminalia* spp.
2		Bairabi	24°18'83"N; 92°53'73"E		
3		Chawngte	22°62'85"N; 92°64'58"E		
4		Tuipui ferry	22°51'45"N; 92°93'73"E		
5		Kawlchaw	22°40'22"N; 92°95'67"E		
6		Tuipui D	22°90'62"N; 92°93'26"E		
7		Vairengte	24°49'10"N; 92°75'68"E		
8	400-800	Nghasih	22°89'49"N; 92°78'34"E	Moist Deciduous Forests & Bamboo Forests	*Lagerstroemiaspeciosa*, *Gmelinaarborea, Albizia* spp., *Melocannabaccifera, Dendrocalamuslongispathus, Bambusatulda*
9		West Phaileng	23°70'48"N; 92°48'82"E		
10		Kolasib	24°22'48"N; 92°67'89"E		
11		Zokhawthar	23°47'95"N; 93°30'86"E		
12		Lawipu	23°42'57"N; 92°39'40"E		
13		Thenzawl	23°28'17"N; 92°77'89"E		
14		Lawngtlai	22°53'01"N; 92°89'88"E		
15	800-1200	Dinthar	23°94'40"N; 92°48'97"E	Subtropical Forests	*Quercus* spp., *Castanopsis* spp., *Schimawallichii, Engelhardtia* spp.
16		Dengsur	23°38'16"N; 92°61'13"E		
17		Tawipui North	22°68'10"N; 92°84'31"E		
18		Haulong	23°05'23"N; 92°77'25"E		
19		Selesih	23°80'68"N; 92°73'32"E		
20		Mampui	22°52'88"N; 92°84'86"E		
21		Lunglei Bazar	22°89'67"N; 92°75'04"E		
22		Rulchawm	23°66'33"N; 92°96'54"E		
23	1200-1600	Hermon veng	23°49'98"N; 93°19'80"E	Subtropical Pine and Montane Forests	*Pinuskesiya, Quercusgriffithii, Rhododendronarboreum, Castanopsistribuloides*
24		Serchhip	23°30'60"N; 92°85'28"E		
25		Hospital Veng	23°47'95"N; 93°30'86"E		
