# Supplementary figures and images for: Alpha diversity with increasing altitude and Rapoport’s rule adherence: Elevational trends in Noctuoidea (Lepidoptera) of Mizoram, India
Source: Biodivers Data J. 2025 Apr 25;13:e152977. doi: 10.3897/BDJ.13.e152977 (PMC12048818; doi:10.3897/BDJ.13.e152977)

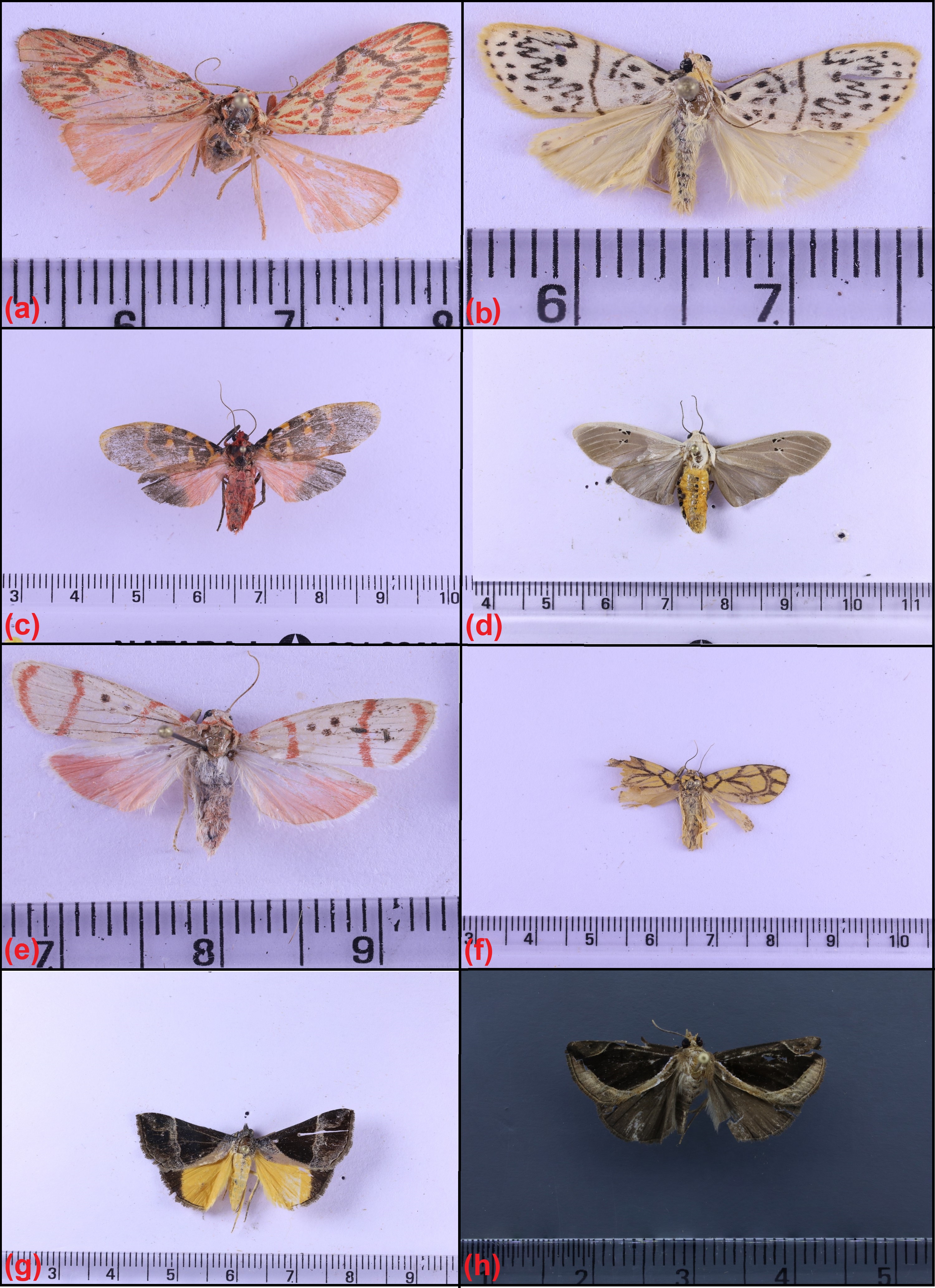

Supplement: Supplementary material 5 — Plates of specimen collected 1 [file bdj-13-e152977-s005.jpg]

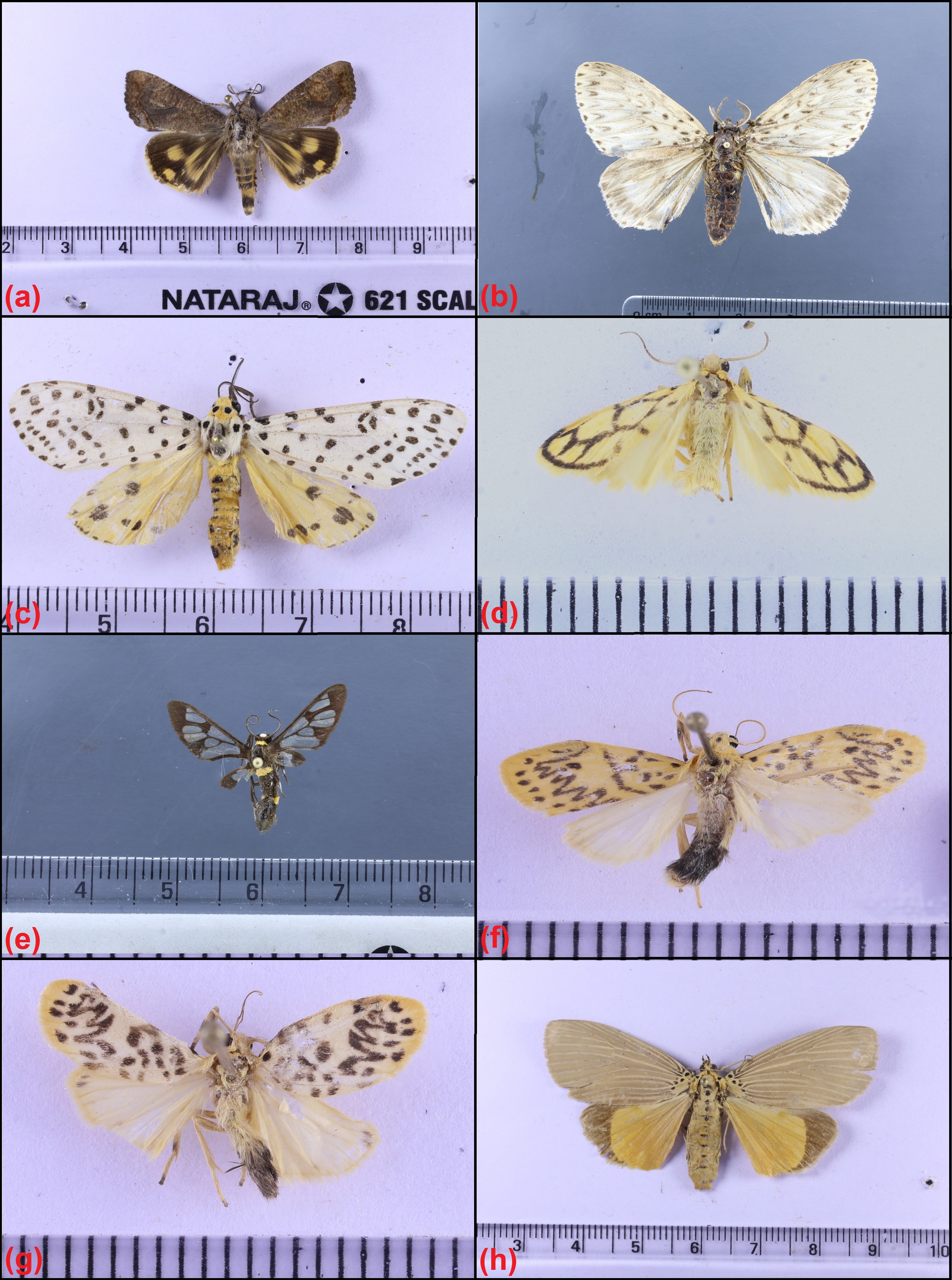

Supplement: Supplementary material 6 — Plates of specimen collected 2 [file bdj-13-e152977-s006.jpg]

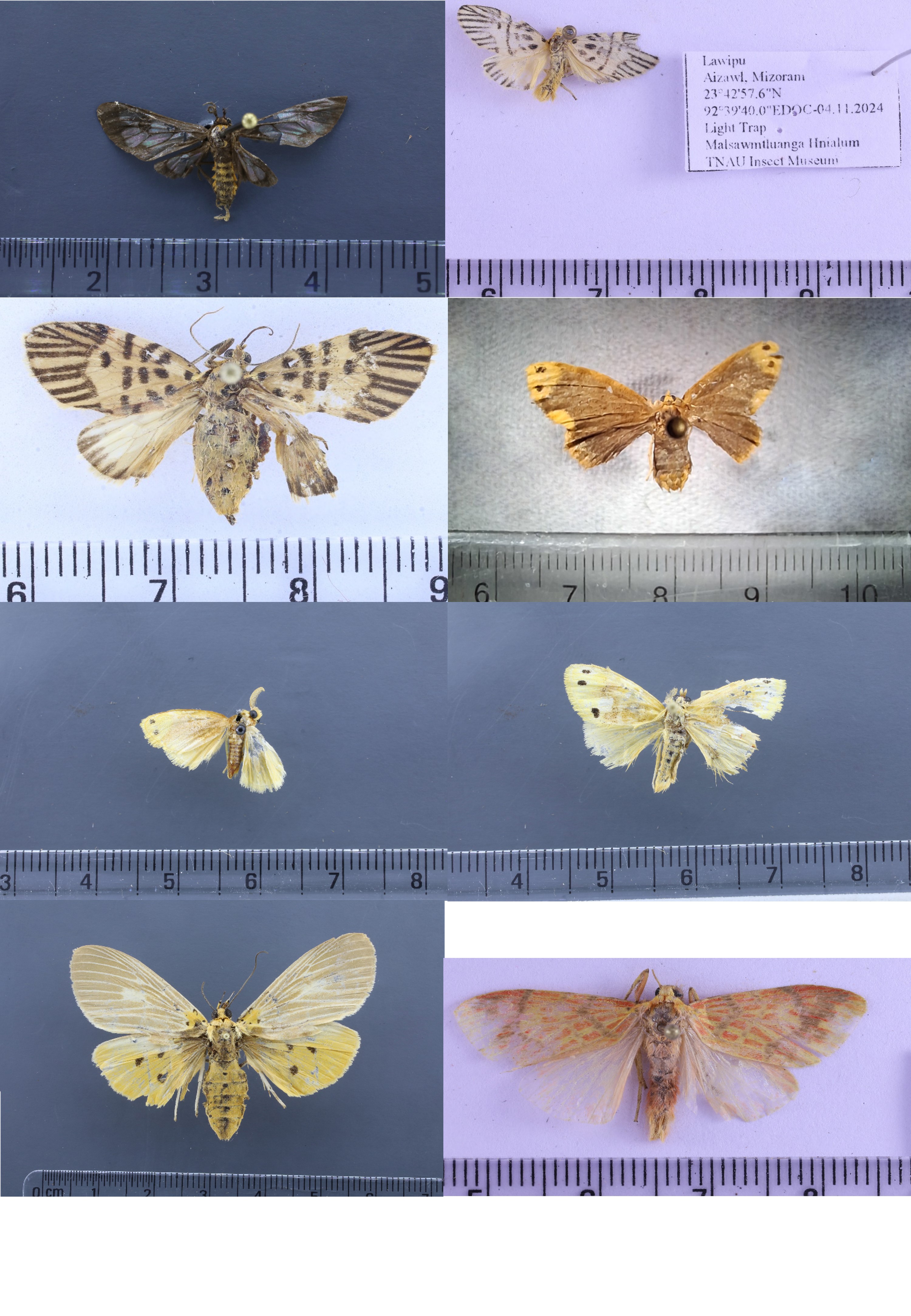

Supplement: Supplementary material 7 — Plates of specimen collected 3 [file bdj-13-e152977-s007.jpg]

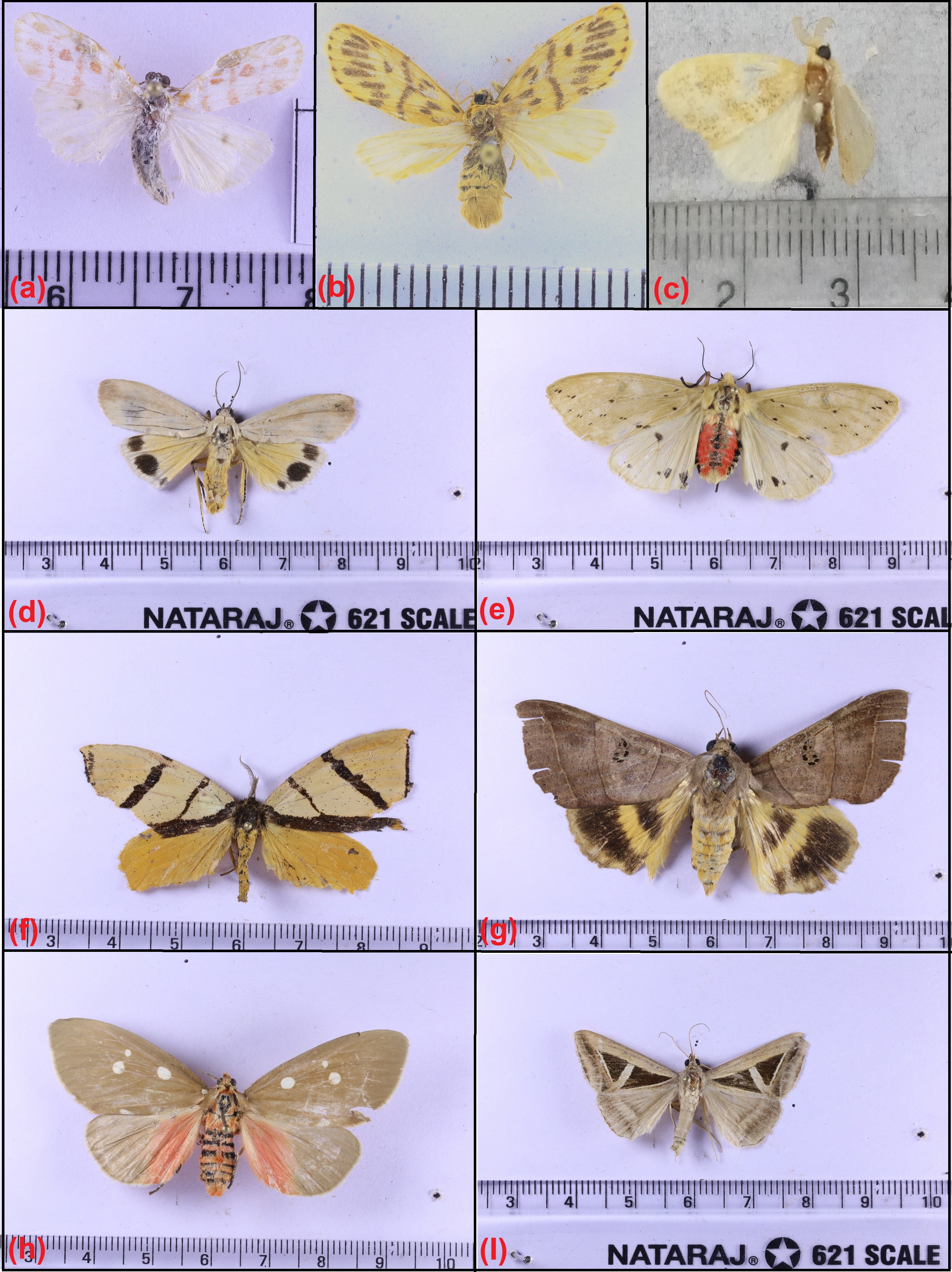

Supplement: Supplementary material 8 — Plates of specimen collected 4 [file bdj-13-e152977-s008.jpg]

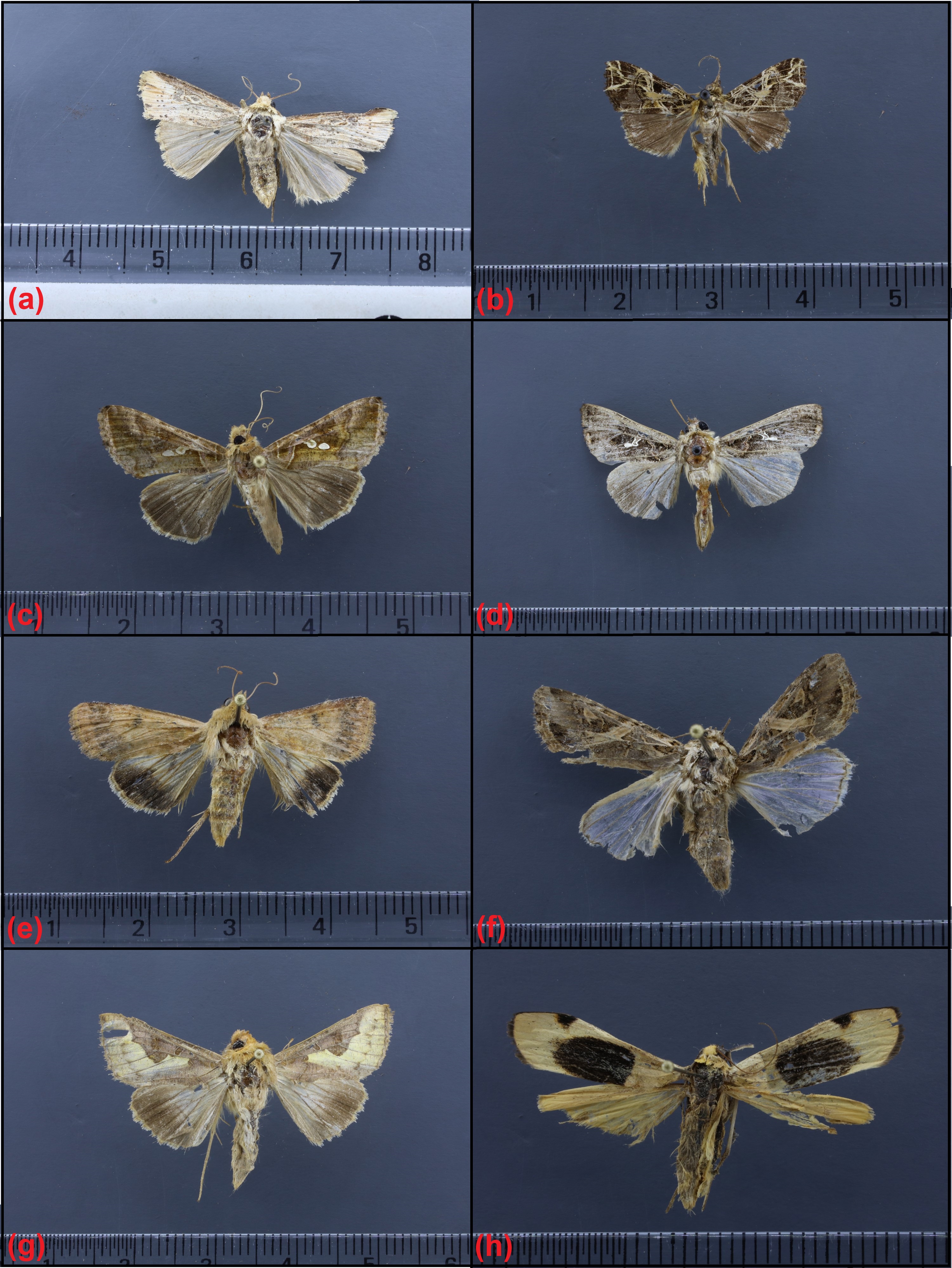

Supplement: Supplementary material 9 — Plates of specimen collected 5 [file bdj-13-e152977-s009.jpg]
